# Mutual regulation of JAG2 and PRAF2 promotes migration and invasion of colorectal cancer cells uncoupled from epithelial–mesenchymal transition

**DOI:** 10.1186/s12935-019-0871-5

**Published:** 2019-06-11

**Authors:** Wan He, Jun Tang, Wenwen Li, Yong Li, Yi Mei, Lisheng He, Keli Zhong, Ruilian Xu

**Affiliations:** 1 Department of Oncology, The Second Clinical Medical College (Shenzhen People’s Hospital), Jinan University, Shenzhen, 518020 Guangdong China; 2 Department of Interventional Radiology, The Second Clinical Medical College (Shenzhen People’s Hospital), Jinan University, Shenzhen, 518020 Guangdong China; 3 Department of Gastroenterology, The Second Clinical Medical College (Shenzhen People’s Hospital), Jinan University, Shenzhen, 518020 Guangdong China; 4 Department of Pathology, The Second Clinical Medical College (Shenzhen People’s Hospital), Jinan University, Shenzhen, 518020 Guangdong China; 5 Department of Gastrointestinal Surgery, The Second Clinical Medical College (Shenzhen People’s Hospital), Jinan University, Shenzhen, 518020 Guangdong China

**Keywords:** Jagged 2 (JAG2), Colorectal cancer, Epithelial–mesenchymal transition (EMT), Notch, PRAF2

## Abstract

**Background:**

Our previous studies revealed that Jagged 2 (JAG2) is involved in the regulation of migration and invasion of colon cancer cells without affecting cell proliferation. This study further explored the specific mechanism by which JAG2 promotes migration and invasion of colorectal cancer cells.

**Methods:**

JAG2 mRNA expression in different clinical stages of colorectal cancer and normal intestinal tissues was detected by quantitative PCR (QPCR). QPCR and Western Blot were used to analyze the differential expression of JAG2 mRNA and protein between normal human colon tissue cells and various colorectal cancer cells. Co-expression status of JAG2 and epithelial–mesenchymal transition (EMT) markers in colon cancer tissues and cells was analyzed. The difference between TGF-β-induced EMT model and the JAG2 overexpression model were compared in promoting migration and invasion of HT29 cells. HT29 cells were treated with EMT pathway inhibitors (LY2157299 and Slug siRNA) to identify a cross-talk between the JAG2 effect and the Notch pathway. Co-expressed genes of JAG2 in colorectal cancer cells were identified using siRNA and transcriptome microarray technology. The mutual regulation of JAG2 and the co-expressed gene PRAF2 and the regulation of the paracrine effect of exosomes were analyzed.

**Results:**

JAG2 was abnormally expressed in colorectal cancer tissues and directly related to clinical stages. Similar to the findings in tissues, the expression of both JAG2 mRNA and protein was significantly increased in the colorectal cancer cell lines compared with that of normal colorectal cell line CCD18-Co. It was shown in our cell model that JAG2 was involved in the regulation of migration and invasion independent of the canonical Notch signaling pathway. More interestingly, JAG2 also promoted the migration and invasion of colon cancer cells in a non-EMT pathway. Further analysis revealed the co-expression of JAG2 with PRAF2 in colorectal cancer cells. JAG2-rich exosomes were released from colorectal cancer cells in a PRAF2-dependent way, while these exosomes regulated the metastasis of colorectal cancer cells in a paracrine manner.

**Conclusions:**

This is the evidence supporting the biological function of JAG2 through non-canonical Notch and non-EMT-dependent pathways and also the first demonstration of the functions of PRAF2 in colorectal cancer cells. These findings also provide theoretical basis for the development of small molecules or biological agents for therapeutic intervention targeting JAG2/PRAF2.

**Electronic supplementary material:**

The online version of this article (10.1186/s12935-019-0871-5) contains supplementary material, which is available to authorized users.

## Background

Cancer invasion and metastasis is one of the leading causes of tumor-related death worldwide and many signaling pathways, including the Hedgehog, Wnt, and Notch, are involved in this process [1]. The Notch signaling pathway is closely related to a broad range of cellular processes, including cell differentiation, proliferation, apoptosis, and adhesion as well as epithelial–mesenchymal transition and it regulates the development of various tissues and organs [2, 3]. Research increasingly shows that abnormal activation of the Notch signaling pathway is implicated in the occurrence, development, invasion, and metastasis of many malignant tumors [4, 5].

The Notch signaling pathway consists of Notch receptors and their ligands. The Notch receptor proteins are a group of type I single-pass transmembrane proteins with a molecular mass of approximately 300 kDa. Mammals possess four homologous notch receptors, referred to as NOTCH1, NOTCH2, NOTCH3, and NOTCH4 [6]. These receptors are expressed as heterodimers on the cell membrane and composed of an extracellular region, a single transmembrane-pass, and an intracellular region [7]. The extracellular domain of Notch (NECD) consists of 29–36 tandem epidermal growth factor-like repeats (EGF-Rs) that bind to the ligands. Following the EGF-Rs is three cysteine-rich Lin-12/Notch repeats (LNRs) that prevent ligand-independent signal transduction. The extracellular domain is the site where the ligand binding and activation of the Notch receptors occur. The Notch intracellular domain (NICD) is comprised of a RAM domain (RBP-J kappa associated molecule), 6 ankyrin (ANK) repeats, 2 nuclear localization sequences (NLS, 1 NLS in Notch4), 1 transactivation domain (TAD; not identified in Notch3 and Notch4), and 1 praline-glutamate-serine-threonine-rich motif (PEST). NICD is the active component of the Notch receptor [8]. The RAM domain is the main binding site for C promoter binding factor-1/Recombination signal binding protein-Jkappa, CBF1/RBPJк); the ANK mediates the interaction between Notch and other proteins and plays an important role in the Notch signaling pathway as an enhancer for the activation of the Notch signal; and the PEST motif is involved in the degradation of the Notch receptors. Notch ligands, including Delta and Serrate in Drosophila and Jagged1 (JAG1), Jagged2 (JAG2), Delta1, Delta3, and Delta4 in human, are also single-pass transmembrane proteins and expressed on adjacent cells [6].

Notch is activated mainly via the canonical and non-canonical pathways [9]. The canonical pathway is a CSL (CBF1 in mammals, Su(H) in Drosophila, and LAGH in *C. elegans*)-dependent Notch signal transduction and the non-canonical pathway is CSL-independent [7]. Notch precursor protein, encoded by the Notch gene, is synthesized in ribosome and then cleaved by a furin-like convertase into two subunits at the S1 site. The two subunits bind together to form a heterodimer, which trafficks to the cell membrane as a mature Notch receptor. Notch receptors bind to their corresponding ligands to activate the Notch signal, and at this moment the Notch receptor protein undergoes two more cleavages. The first cleavage is catalyzed by the TNF-α converting enzyme (TACE) of the ADAM protease family at the S2 site in the extracellular domain to release NECD. The second one is by the γ- secretase at the S3 site in the transmembrane region close to the membrane. The NICD is released and translocated into the nucleus where its biological function is achieved. In the canonical Notch pathway, RAM23 region binds to CSL after the nucleus translocation of NICD. If the Notch signaling pathway is inactivated, CSL binds to the promoter of Notch target genes and corepressor (CoR) is recruited to repress the expression of the target genes. Once NICD binds to CSL in the nucleus, coactivator (CoA) is recruited to replace the CoR, and thereby the transcription of the target genes is activated. A few Notch target genes have been currently identified.

The human JAG2 gene is located on chromosome 14q32 and consists of 26 exons which encode mRNA variants with 5825 bp and 5721 bp in length (GenBank NM_002226.4 and NM_145159.2). The protein encoded by this gene contains a signal peptide, an extracellular DSL region, 16 EGF-like repeats, a cysteine-rich region, a transmembrane region, and a short cytoplasmic region of 132 amino acids [10]. Studies have shown that JAG2 promotes tumor cell metastasis in various tumors [11–14]. Xing et al. [15] reported that the metastasis-free survival and overall survival rates in 779 breast cancer patients are associated with JAG2, one of the five canonical NOTCH ligands, and hypoxia-induced tumor invasion and activation of JAG2 in normal bone marrow stroma plays an important role in tumor progression and metastasis. Therefore, JAG2 can be considered as a prognostic indicator and as a new therapeutic target for metastatic breast cancer. It was found in a recent study that the expression of JAG2 protein is elevated in metastatic medulloblastoma tissues and associated with a poor prognosis, and the proliferation rate and migration ability of medulloblastoma cell lines are reduced by siRNA-mediated JAG2 gene silence [11].

Our previous studies showed that JAG2 was highly expressed in colorectal cancer cells and the migration and invasion ability, rather than the proliferation ability, of the cells was affected by the altered relative expression level of JAG2 [16]. In the present study, the regulation mechanism of JAG2 on cell migration was further explored, and the pathways involved were different from the canonical Notch signaling pathway. These results contribute to the further analysis of the mechanisms involved in the metastasis of colorectal cancer.

## Materials and methods

### Clinical specimens

A total of 72 snap-frozen normal and CRC specimens were collected at the Shenzhen People’s Hospital (Shenzhen, China). The tissues collected included 20 normal tissue sections and 52 CRC tissues, including 20 N0, 19 N1 and 13 N2 CRC samples. This study was approved by the Shenzhen People’s Hospital, Second Clinical Medical College of Jinan University, and written informed consent was obtained from all participants.

### Cell culture

The human colorectal cancer cell lines, including RKO, HT29, HCT116, SW620, SW480 and DLD-1, were obtained from the Cell Resource Center, Peking Union Medical College. The cells were cultured in DMEM (Invitrogen, Carlsbad, CA, USA) supplemented with 10% (v/v) fetal bovine serum (FBS; Invitrogen), 100 U/mL penicillin G, and 100 mg/mL streptomycin sulfate (Sigma-Aldrich) at 37 °C with 5% CO_2_. CCD-18co human normal fibroblast cell line (ATCC, CRL-1459) were grown in MEM supplemented with 10% fetal bovine serum, 1% antibiotic/antimycotic, 2 mM GlutaMAX, 0.1 mM Non-essential amino acids (NEAA) (Invitrogen) and 0.57 mM Recombinant Human TNF-α (Peprotech, USA).

### Establishment of EMT model

HT29 cells were cultured in DMEM containing 10% fetal bovine serum and 100 U/mL streptomycin. TGF-β (PeproTech Inc. Rocky Hill, USA) was added to the culture medium at the final concentration of 5 ng/mL. 24 h later, the cells were harvested for subsequent detection.

### Cell transfection and treatment

Cells were plated in 6-well or 24-well plates and transfected with siRNA (Ruibo Bio, Guangzhou, China) or vectors using Lipofectamine 2000 (Invitrogen) according to the manufacturer’s recommendations. Transfected cells were used in further assays or RNA/protein extraction. The plasmid pCMV-JAG2 was obtained from OriGene (OriGene Technologies, USA). In TGF-β induced EMT model, cells were treated with 5 ng/mL TGF-β1 combined with 100 nm LY2157299 (Selleck, Houston, USA) or 20 nm Slug siRNA for 24 h. The siRNAs were purchased from Shanghai GenePharma Co., Ltd (China), and all of them were verified by off-target and validity. The siRNA sequences were as follows: JAG2 siRNA: 5′-ACCUGAACUACUGUGGCAGCCdTdT-3′ (sense); PRAF2 siRNA: 5′-CCUCACCCCAAUGUUCCACAdTdT-3′ (sense); NOTCH1 siRNA: 5′-AGCAUCACCUGCCUGUUAGGCdTdT-3′ (sense); NOTCH2 siRNA: 5′-GAUGAUGACGACUUGAAACdTdT-3′ (sense); NOTCH3 siRNA: 5′-GUCUGCAAGGACCGAGUCAAdTdT-3′ (sense); NOTCH4 siRNA: 5′-ACGUAACCACUGGGAUCUGCdTdT-3′ (sense); Slug siRNA: 5′-GAGCAUACAGCCCCAUCACUdTdT-3′ (sense).

### RNA extraction and quantitative real-time PCR analysis

Total RNA was isolated using TRIzol Reagent (Invitrogen). The concentrations of RNA samples were quantified using spectrophotometry (GeneQuant, GE Healthcare, Piscataway, USA) at 260 nm and reverse transcribed to produce cDNA using miScript RT II Buffer (Qiagen). The mix was incubated at 37 °C for 60 min, followed by 95 °C for 5 min. Real-time PCR was performed using miScript HiSpec Buffer (QIAGEN Inc., UK) and ABI 7500 Real-Time PCR System (Applied Biosystems, Warrington, UK). The mixtures were incubated at 95 °C for 15 min, followed by 40 cycles of 94 °C for 20 s, 55 °C for 20 s and 70 °C for 30 s. Gene expression was measured in triplicate and data were processed using 2^−ΔΔCT^ method and normalized to control. GAPDH was used as the endogenous control. The primers sequences used for RT-qPCR were as follows:

JAG2: 5′-AGCCATGCCTTAACGCTTTT-3′ (forward), 5′-CACACACTGGTACCCGTTCA-3′ (reverse); PRAF2: 5′-ACGACTTTGTTCTGGGGTCG-3′ (forward), 5′-GATGCCGAAGCAGAGAAGGT-3′ (reverse); NOTCH1: 5′-GGACGTCAGACTTGGCTCAG-3′ (forward), 5′-ACATCTTGGGACGCATCTGG-3′ (reverse); NOTCH2: 5′-CCAGGAGAGGTGTGCTTGTT-3′ (forward), 5′-TCTTCACAGAGTAGGCCCCG-3′ (reverse); NOTCH3: 5′-CAGGGACGTCAGTGTGAACT-3′ (forward), 5′-TTGGTGCAGATACCATGAGGG-3′ (reverse); NOTCH4: 5′CCCATTAAAAGGCAGGCTGGA-3′ (forward), 5′-TGCCTGCAATTCTTGGTTCC-3′ (reverse); SLUG: 5′-CCCTCACTGCAACAGAGCAT-3′ (forward), 5′-TGCTACACAGCAGCCAGATT-3′ (reverse); GAPDH: 5′-ACAACTTTGGTATCGTGGAAGG-3′ (forward), 5′-GCCATCACGCCACAGTTTC-3′ (reverse).

### Protein extraction and western blot analysis

Total cell lysates from cells were prepared using ProteoJET™ Mammalian Cell Lysis Reagent according to the manufacturer’s instructions (Thermo Scientific, Rockford, IL, USA), and protein concentrations were determined using the BCA method. Specific antibodies to JAG2 (ab109627), PRAF2 (ab53113), NICD (ab83232), E-cadherin (ab1416), vimentin (ab92547), CD63 (ab59479), CD81 (ab79559), NOTCH1 (ab52627), NOTCH2 (ab8926), NOTCH3 (ab23426), NOTCH4 (ab184742) and β-actin (ab8226) were purchased from Abcam (Cambridge, UK).

### Migration and invasion assays

Migration and invasion assays of CRC cells were conducted using 24-well Transwell chambers (8-μm pore size polycarbonate membrane; Costar, Corning, NY). For the migration assays, 5 × 10^3^ cells were suspended in 100 μL DMEM and seeded into the upper chamber; 600 μL DMEM containing 10% FBS was added to the outside chamber. After incubating at 37 °C under 5% CO_2_ for 24 h, the upper chamber was removed and cells that had not migrated were wiped with a cotton swab. After fixing with 4% paraformaldehyde, nuclei were stained with 4′,6-diamidino-2-phenylindole (DAPI). Images of different fields were collected, and statistical analyses performed. For invasion assays, Matrigel (80 μg/mL, BD Biosciences) was pre-loaded in the upper chamber, and the remaining steps were performed as outlined for the migration assays. In each experiment, the number of cells in five random fields on the underside of the filter was calculated, and three independent filters were analyzed.

### Proteomics sample preparation

The treated cells were lysed immediately with the compatible lysis buffer containing 10 mM HEPES, pH 7.4, 150 mM NaCl, 2 mM CaCl2, 2 mM MgCl2, 600 mM guanidine HCl, 1% DDM, and protease inhibitor mixture (1 mM EDTA, 1 mM PMSF, 1 μg/mL leupeptin, 1 μg/mL pepstatin, and 1 μg/mL aprotinin). Protein concentration was determined by Pierce Micro BCA Kit (Thermo). The obtained tissue lysate was processed by using SISPROT protocol as previously described [17]. Briefly, the samples were firstly acidified to pH 2–3 and loaded onto 200 μL or 10 μL spintip device packed with one plug of C18 disk (3 M Empore, USA) and 0.6 mg of 20 μm POROS SCX beads (Applied Biosystems, USA) in tandem. Proteins were reduced by TCEP, alkylated by IAA and digested by trypsin (TPCK-treated, Sigma-Aldrich). The digested peptides were then transferred from the SCX beads to C18 disk with 200 mM ammonium formate (pH 10) and eluted from C18 disk with ACN concentration of 80% in 5 mM ammonium formate (pH 10).

### Nano-LC–MS/MS analysis

The obtained samples were resuspended in 0.1% (v/v) formic acid (FA) and analyzed by a Q-Exactive mass spectrometer coupled with an Easy-nLC 1000 (ThermoFisher Scientific). The LC separation was performed with an integrated spraytip column (100 μmi.d. × 20 cm) packed with 1.9 μm/120 Å ReproSil-Pur C18 resins (Dr. Maisch GmbH, Germany). The gradient solvent system consisted of solvent A [0.1% (v/v) FA in water] and solvent B [0.1% (v/v) FA in ACN]. 80% (v/v) of the peptide samples were loaded and separated at a flow rate of 250 nL/min. The solvent B was changed linearly as follows: 0 min, 3%; 2 min, 7%; 52 min, 22%; 62 min, 35%; 64 min, 90%; 70 min, 90%; 72 min, 3%; 80 min, 3%. Full MS scans were performed in mass analyzer over m/z range of 350–1550 with a mass resolution of 120,000. The MS/MS spectra were acquired in data-dependent mode with a 3 s Top Speed method. Tandem MS was performed in the ion trap mass analyzer using an isolation window of 1.6 Da by quadrupole mass analyzer and HCD fragmentation with normalized collision energy of 30. The dynamic exclusion time was set as 60 s.

Raw data were searched against the human Uniprot fasta database (70,332 entries, downloaded on Sep 29, 2016) using MaxQuant (version 1.5.5.1). A maximum of two missed cleavages was allowed. Cysteine carbamidomethylation was set as fixed modification, while methionine oxidation, asparagine, and glutamine deamidation were set as variable modifications. FDR was set to 0.01. Statistical analyses were performed with the Perseus software (version 1.5.5.3) [18].

### Co-expression analysis

The starBase (http://starbase.sysu.edu.cn/) is a database that deciphers the regulation relationships between RNA and RNA, or protein and RNA by managing the 108 CLIP-Seq datasets from 37 independent studies. It was used to explore positive-correlation (Pearson correlation: r < 0, P-value < 0.05) between mRNA and mRNA across colon and rectal cancer.

### Exosomes extraction

Exosomes in the culture supernatant were prepared using the ExoQuick-TC kit (System Biosciences, Mountain View, CA, USA) as described by the manufacturer. In general, the medium was centrifuged for 15 min at 3000×*g* to remove apoptotic cells and cell debris. After adding 3.3 mL of the exosome-precipitating solution to each 10 mL of the culture supernatant, the cells were refrigerated overnight, and then the mixed liquid was centrifuged at 10,000×*g* for 30 min, and the supernatant was discarded; the separated exosomes were suspended in PBS, stored at − 80° C or used directly. Total RNA and protein in exosomes were isolated as the methods described above in cells.

### Quantification of exosomes

Relative quantification of exosomes was performed using the EXOCET Exosome Quantitation Kit (System Biosciences). Basic procedure: A standard curve was prepared using exosome standards provided in the kit. Add 20 μL of exosomes suspension to 80 μL lysis Buffer, incubate at 37 °C for 5 min, centrifuged at 1500×*g* for 5 min, and incubate the supernatant on ice. 50 μL of the reaction solution was added to 50 μL of the supernatant, and the absorbance was measured at 405 nm after 20 min at room temperature. The number of exosomes was calculated from the standard curve.

### Immunofluorescent analysis

HT29 cells were treated with or without exosomes. The cells were permeabilized in 0.1% Triton X-100 and blocked with 5% bovine serum albumin. All cells were then fixed with 4% paraformaldehyde and incubated with primary antibody anti-JAG2 (Abcam, ab109627) overnight at 4 °C. FITC-labeled secondary antibody (1:200 dilutions, BOSTER, BA1127) was added for 2 h at 37 °C. DAPI reagent was used to stain the HT29 cell nuclei. Image acquisition was done with Olympus FV1000 confocal microscope.

### Statistical analysis

All experiments were performed in triplicate. All data were analyzed using SPSS 19.0 statistics software (IBM). Analysis of variance (ANOVA) was used to evaluate the statistical difference between groups. *P*-values < 0.05 were considered statistically significant.

## Results

### Abnormal expression of JAG2 in colorectal cancer tissues and cells

**Fig. 1:**
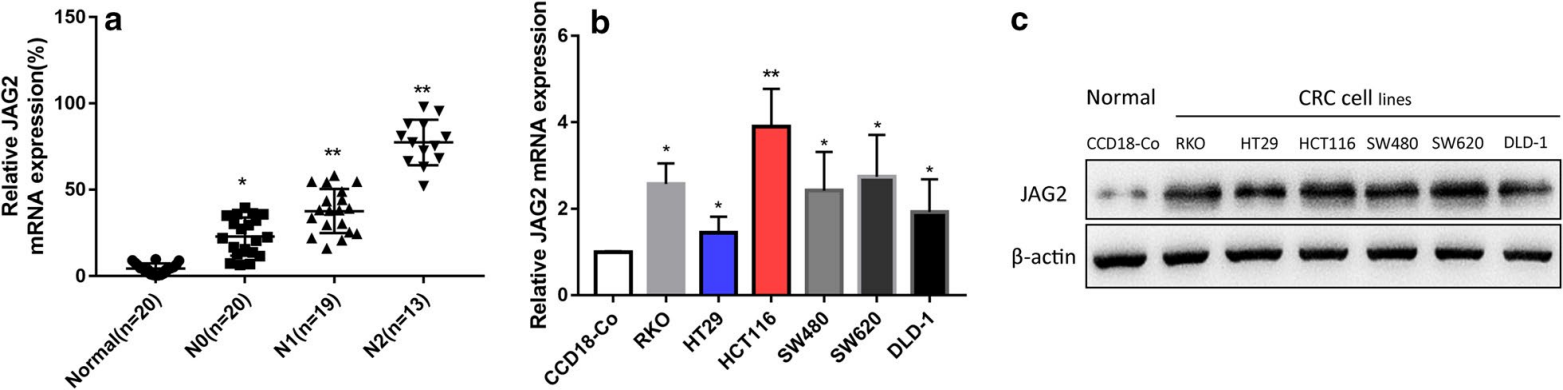
Analysis of JAG2 expression in colorectal cancer tissues and cells. **a** Quantitative PCR analysis showed the expression of JAG2 mRNA in COAD tissues was increased with the clinical stages. GAPDH served as an internal control. **b** Quantitative PCR analysis showed that JAG2 mRNA was significantly increased in colorectal cancer cells compared with that of the normal colorectal cells. GAPDH served as an internal control. **c** Western blot analysis showed that JAG2 protein was significantly increased in colorectal cancer cells compared with that of the normal colorectal cells. β-actin served as an internal control. *P < 0.05 and **P < 0.01. Data are representative of three independent experiments (mean ± S.D.)

First, the expression of JAG2 in colorectal cancer tissues was confirmed. The relative expression of JAG2 mRNA in colorectal cancer tissues was determined by quantitative PCR and the results showed that the overall expression of JAG2 in colorectal cancer tissues was increased compared with that of adjacent tissues and the relative content of JAG2 mRNA increased with the clinical stages (N0, N1, and N2) (Fig. 1a), indicating that JAG2 was abnormally expressed in colorectal cancer tissues and directly related to clinical stages of the disease.

Next, the differential expression of JAG2 in normal colorectal cell line CCD18-Co and colorectal cancer cell lines (RKO, HT29, HCT116, SW620, SW480 and DLD-1) was then compared. Similar to the findings in tissues, the expression of both JAG2 mRNA and protein was significantly increased in these colorectal cancer cell lines compared with that of the CCD18-Co (Fig. 1b, c).

### JAG2 increased cell migration and invasion uncoupled from EMT

**Fig. 2:**
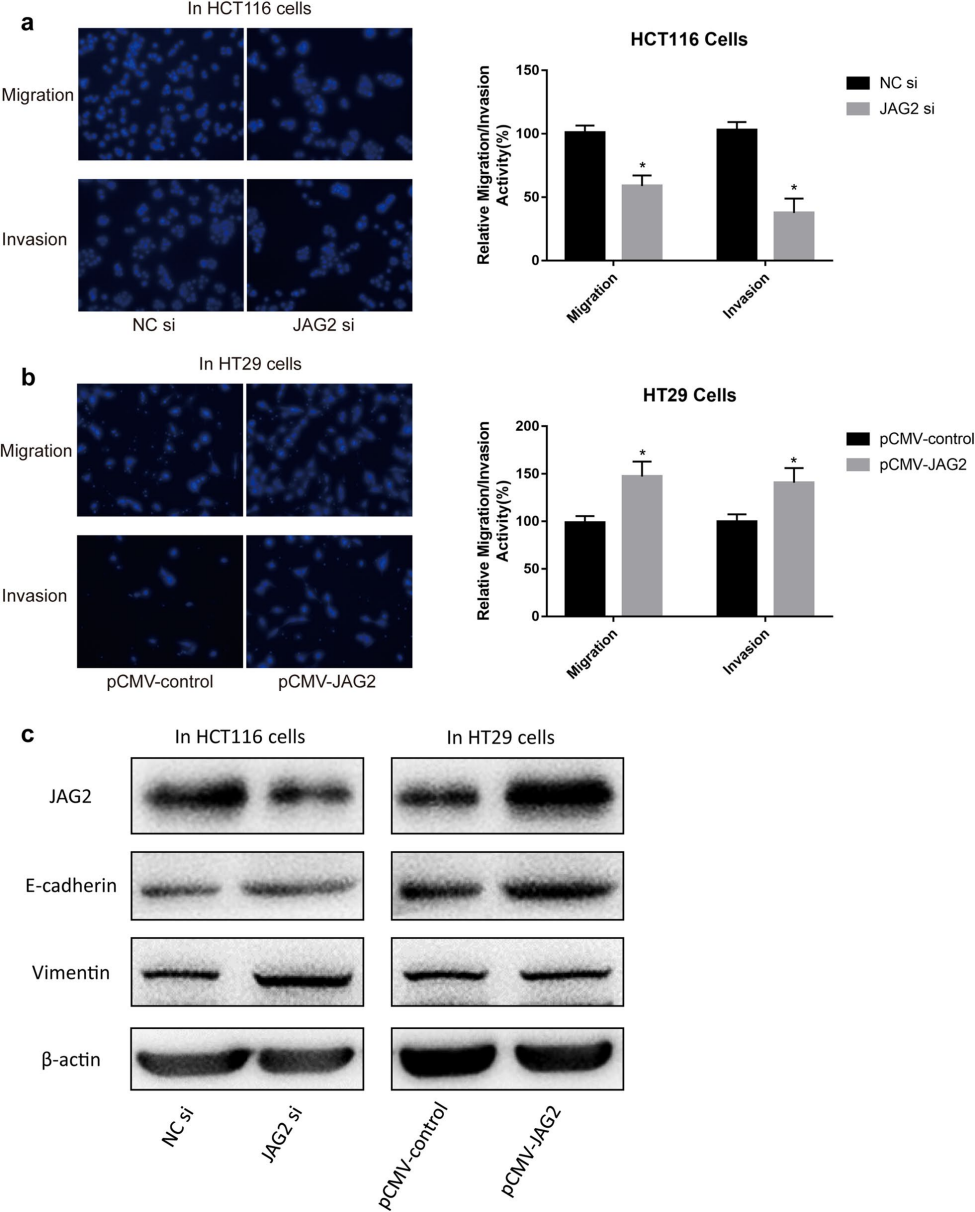
Effect of different relative expression of JAG2 on the migration and invasion ability of colorectal cancer cell line HT29 and its correlation with the EMT pathway. **a** JAG2 siRNA inhibited the migration and invasion of human colorectal cancer cells. Migration and invasion assay of HCT116 cells following transfection with NC siRNA or JAG2 siRNA using the transwell apparatus. Microscopic analysis of migrated or invaded cells stained with DAPI (left). Relative cell migration or invasion was determined by the number of the DAPI-stained cells (right). Cell migration or invasion is expressed as a percentage of that observed in control. **b** JAG2 overexpression promoted the migration and invasion of human colorectal cancer cells. Migration and invasion assay of HT29 cells following transfection with pCMV-control or pCMV-JAG2 vectors. **c** No significant changes of EMT markers were detected with the altered JAG2 expression in human colorectal cancer cells. JAG2 and EMT markers (E-cadherin and vimentin) protein levels were examined by Western Blot analyses in HCT116 and HT29 cells treated with JAG2 siRNA or PCMV-JAG2 vectors. β-actin served as an internal control. Data are the mean ± SD. of three independent experiments. *P < 0.05 versus control

Based on the above results, cell line HCT116 with relatively high JAG2 expression and HT29 with relatively low JAG2 expression were selected for subsequent experiments. Cell migration and invasion were significantly inhibited in HCT116 cells when JAG2 expression was reduced by siRNA (Fig. 2a). On the contrary, cell migration and invasion were significantly increased when JAG2 was overexpressed in the HT29 cells (Fig. 2b). However, no significant changes in EMT markers, including E-cadherin and vimentin, were detected in HCT116 and HT29 cells, although the migration and invasion ability of both cell lines was changed due to the altered JAG2 expression (Fig. 2c). These results suggested that JAG2 was involved in the invasion and migration of colorectal cancer cells and a specific EMT-independent mechanism might present to mediate the metastasis of cancer cells.

**Fig. 3:**
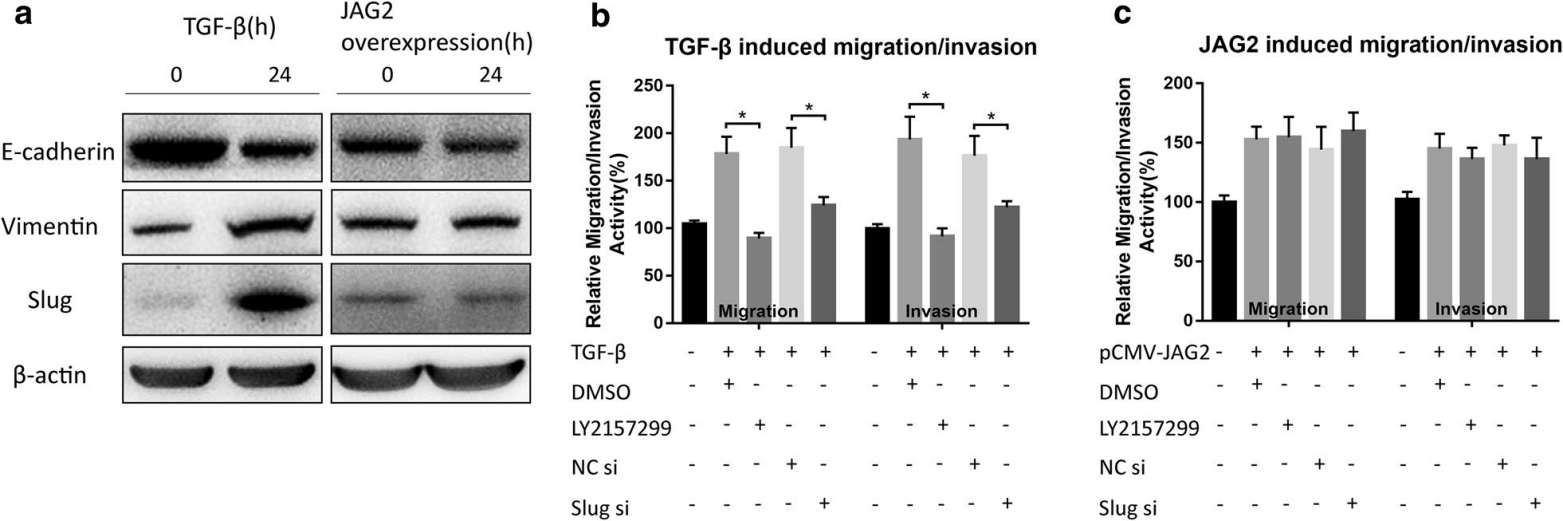
Comparison of the TGF-β-induced EMT model and the JAG2-overexpressing cell model. **a** Western blot analysis of the expression of EMT-related markers in HT29 cells after TGF-β induction and JAG2 overexpression. **b** Relative cell migration or invasion abilities were determined in HT29 cells treated with TGF-β (5 ng/mL), LY2157299 (100 nm) or Slug siRNA (20 nm) for 24 h. LY2157299 and Slug siRNA could inhibit TGF-β induced migration and invasion of colorectal cells. **c** Relative cell migration or invasion abilities were determined in JAG2-overexpressed HT29 cells treated with LY2157299 or Slug siRNA. LY2157299 and Slug siRNA could not inhibit JAG2-induced cell migration and invasion. Data are the mean ± SD. of three independent experiments. *P < 0.05 versus control

To further confirm our hypothesis, the difference in cell migration and invasion between the classical TGF-β-induced EMT model and the JAG2 overexpressing cell model was compared. In cellular EMT model of HT29, EMT marker E-cadherin was down-regulated, vimentin and Slug were up-regulated (Fig. 3a), and the enhanced cell migration and invasion ability was significantly inhibited by their corresponding inhibitors (LY2157299 and Slug siRNA) (Fig. 3b). However, these inhibitors were not effective in inhibiting the JAG2-induced cell migration and invasion (Fig. 3c).

**Fig. 4:**
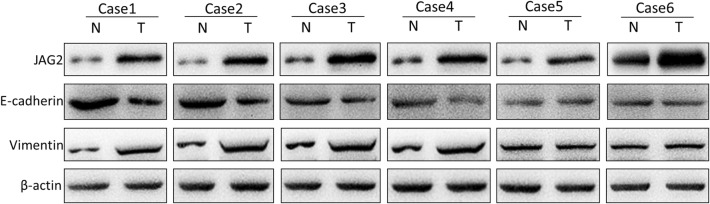
Correlation analysis between JAG2 and EMT markers (E-cadherin and vimentin) in 6 specimens from N1/N2 stage colorectal cancer tissues by Western blot. JAG2 and EMT markers showed marked heterogeneity in expression in colorectal cancer tissues

Co-expression of JAG2 and EMT markers was randomly detected in 6 specimens of N1/N2 colorectal cancer tissues. The results revealed a heterogeneous co-expression pattern of JAG2 and EMT markers, i.e., in 2 of the 6 tissues, JAG2 was highly expressed in and no difference in the expression of EMT markers was found between the cancer tissues and the adjacent tissues (Fig. 4). Undoubtedly, these results further confirmed that JAG2 might promote cell metastasis via inducing EMT-independent signaling pathways.

### JAG2 promoted cell migration and invasion independent of NICD

**Fig. 5:**
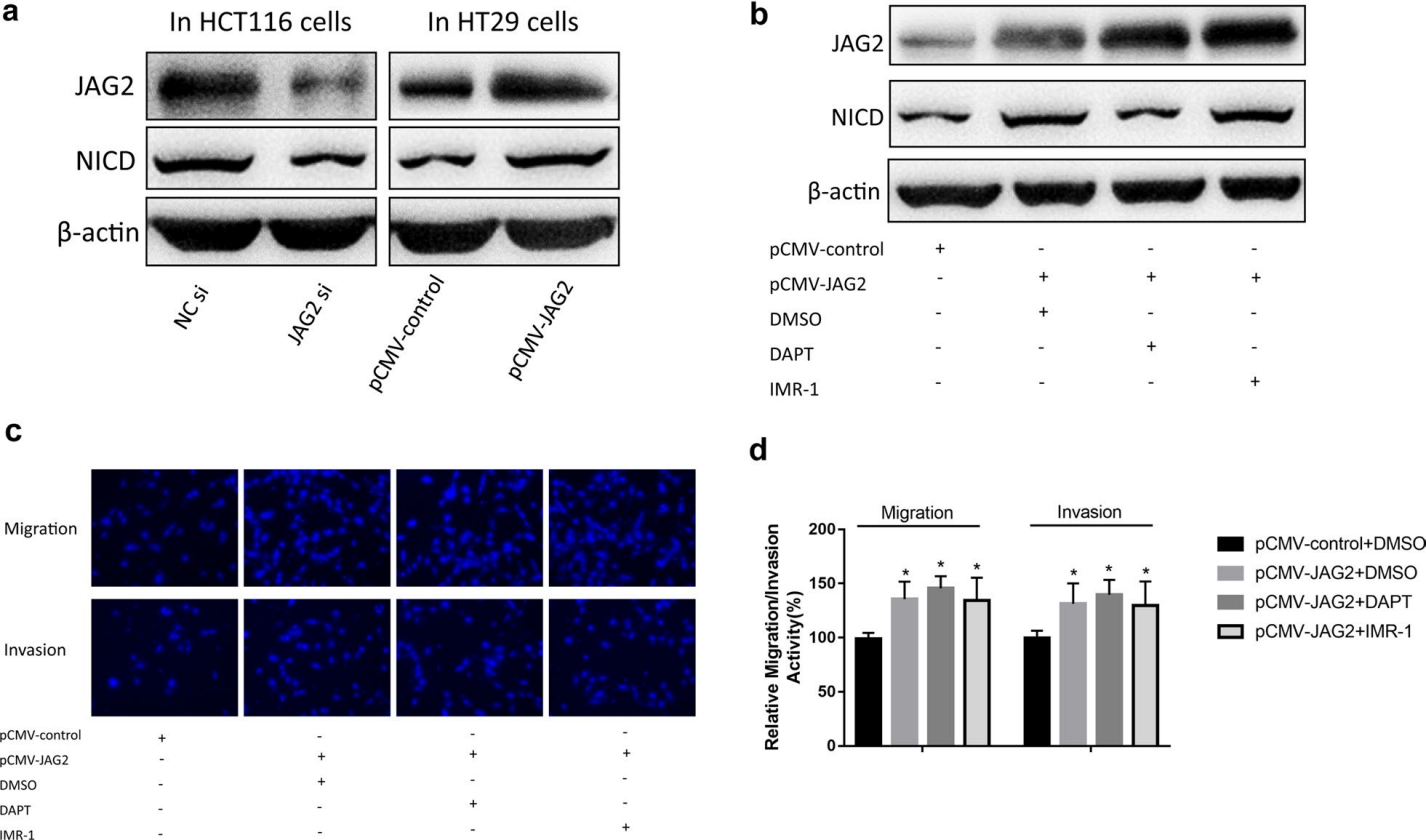
No significant correlation between JAG2-induced cell invasion and migration and the canonical Notch signaling pathway. **a** When JAG2 was silenced or overexpressed in HCT116 and HT29 cells respectively, Western blot analyses showed that NCID protein levels were affected by altered expression level of JAG2. **b** When JAG2 was overexpressed in HT29 cells, JAG2 and NCID protein levels were examined by Western Blot analyses in JAG2-overexpression HT29 cells treated with DAPT (5 μM) or IMR-1 (1 μM). **c**, **d** Cell migration and invasion enhanced by JAG2-overexpression was not effectively repressed by Notch signaling pathway inhibitors (DAPT and IMR-1). Data are the mean ± SD. of three independent experiments. *P < 0.05 versus control

As a canonical ligand for the Notch pathway, JAG2 theoretically binds to the Notch receptor and then NICD is released into the nucleus to exert its biological function. The relative level of NICD was decreased in the HCT116 cells when JAG2 expression was knocked down by siRNA and increased in the HT29 cells when JAG2 was overexpressed (Fig. 5a), indicating that JAG2 did participate in the production and release of NICD. Next, JAG2 was overexpressed in HT29 cells while inhibitors for the Notch signaling pathway (DAPT, a γ-secretase inhibitor and IMR-1, a novel Notch inhibitor targeting the transcriptional activation process) was applied at the same time, and the results showed that DAPT, rather than IMR-1, effectively reduced the relative level NICD (Fig. 5b). Interestingly, application of both inhibitors did not effectively inhibit the increase in JAG2-induced cell migration and invasion (Fig. 5c). Together, these results suggested that although binding of JAG2 to Notch receptors promoted the production and release of NICD, pathways other than the canonical Notch signaling pathway were involved in enhancing cell migration and invasion.

### Co-expression and mutual regulation of PRAF2 and JAG2 in colorectal cancer cells

**Fig. 6:**
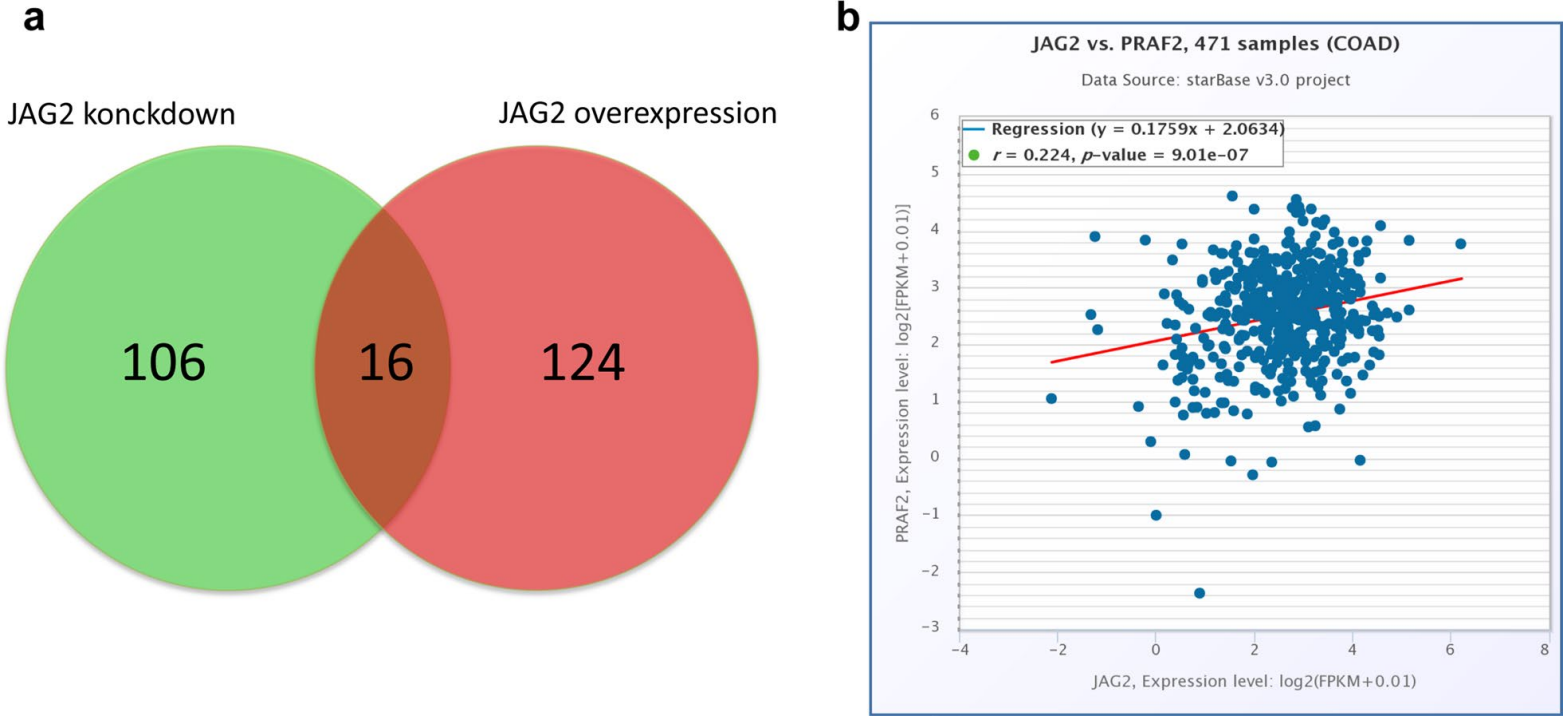
Genes co-expressed with JAG2 in colorectal cancer cells. **a** Venn diagram of differentially expressed proteins in the HCT116 cells with up-regulation or down-regulation of the JAG2. Red indicates the number of proteins that were significantly up-regulated with overexpression of JAG2, and green indicates the number of proteins that were significantly down-regulated with JAG2 knockdown. **b** Co-expression of JAG2 and PRAF2 in colorectal cancer tissues using the Starbase database

**Fig. 7:**
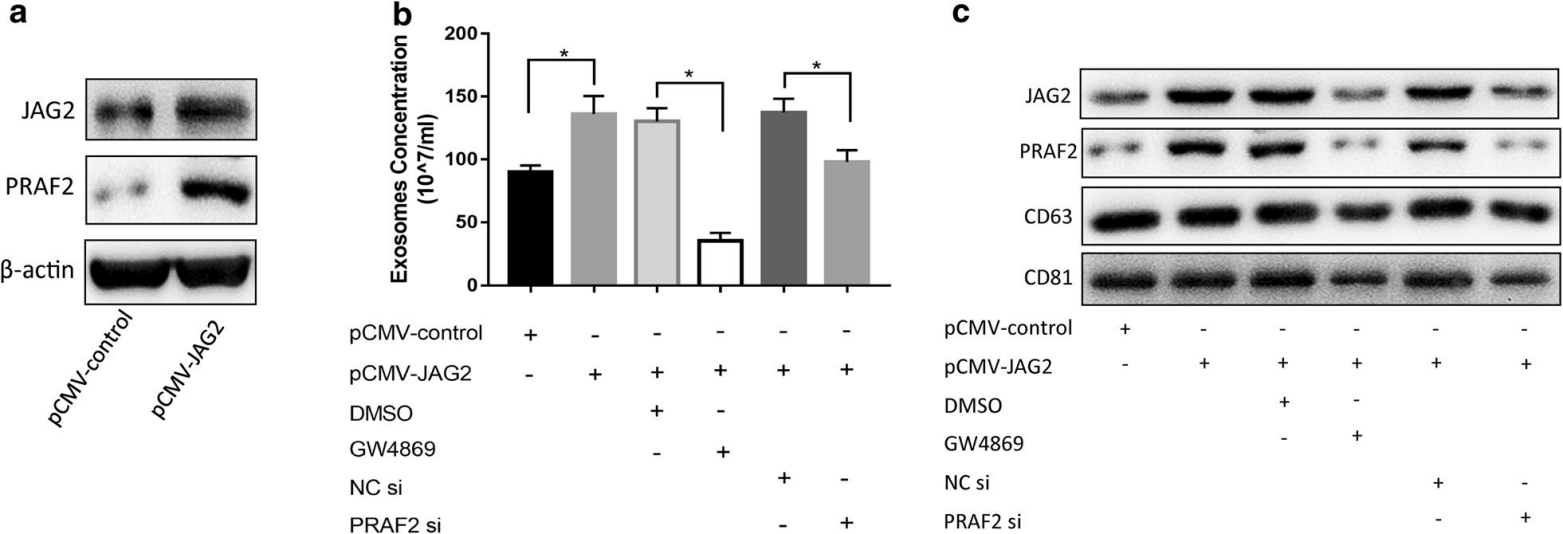
Overexpression of JAG2 in HT29 cells affects the characteristics of exosomes. **a** Western blot analyses showed that overexpression of JAG2 up-regulated PRAF2 protein level; **b** altered JAG2 (overexpression of JAG2) and PRAF2 (downregulation of PRAF2 by siRNA) proteins in HT29 cells affected the number of secreted secretions; **c** when JAG2 was overexpressed in HT29 cells, and PRAF2 was silenced by siRNA or GW4869 (2.5 μM) treatment, Western Blot detected a decrease in the content of JAG2 and PRAF2 in exosomes compared with the control group. Data are the mean ± SD. of three independent experiments. *P < 0.05 versus control

Data from the present study revealed that the ability of JAG2 to promote the migration of colorectal cancer cells was independent of the EMT and the canonical Notch signaling pathway. To answer the questions which signaling pathways are activated by JAG2 and which key factors are affected, genes co-expressed with JAG2 were analyzed. When JAG2 was overexpressed or knocked down in HCT116 cells, a total of 16 proteins with significant expression differences with up- or down-regulation of JAG2 were screened via high-throughput proteomic analysis by bio-mass spectrometry (Fig. 6a), including dynein, enzyme, transporter, adhesion protein, transcriptional regulator, ribosomal protein and collagen binding protein, etc. (Additional file 1: Table S2). Meanwhile, the starBase analysis platform was used to retrieve data from the TCGA database for JAG2 co-expression analysis, and finally PRAF2 was identified (Fig. 6b). The function of PRAF2 is poorly understood and possibly related to vesicle transport. When JAG2 was overexpressed in colorectal cancer cell line HT29, PRAF2 was up-regulated (Fig. 7a), then the number of exosomes secreted by colorectal cancer cells was significantly increased (Fig. 7b) and the level of JAG2 and PRAF2 proteins in exosomes was upregulated (Fig. 7c). These JAG2-overexpression induced effects were effectively inhibited by PRAF2 siRNA and exosome inhibitor GW4869 [19] (Fig. 7b, c). These results suggested that JAG2 promoted the expression of PRAF2, which in turn enhanced the secretion of JAG2 in the form of exosomes.

### Paracrine effect of JAG2-rich exosomes

**Fig. 8:**
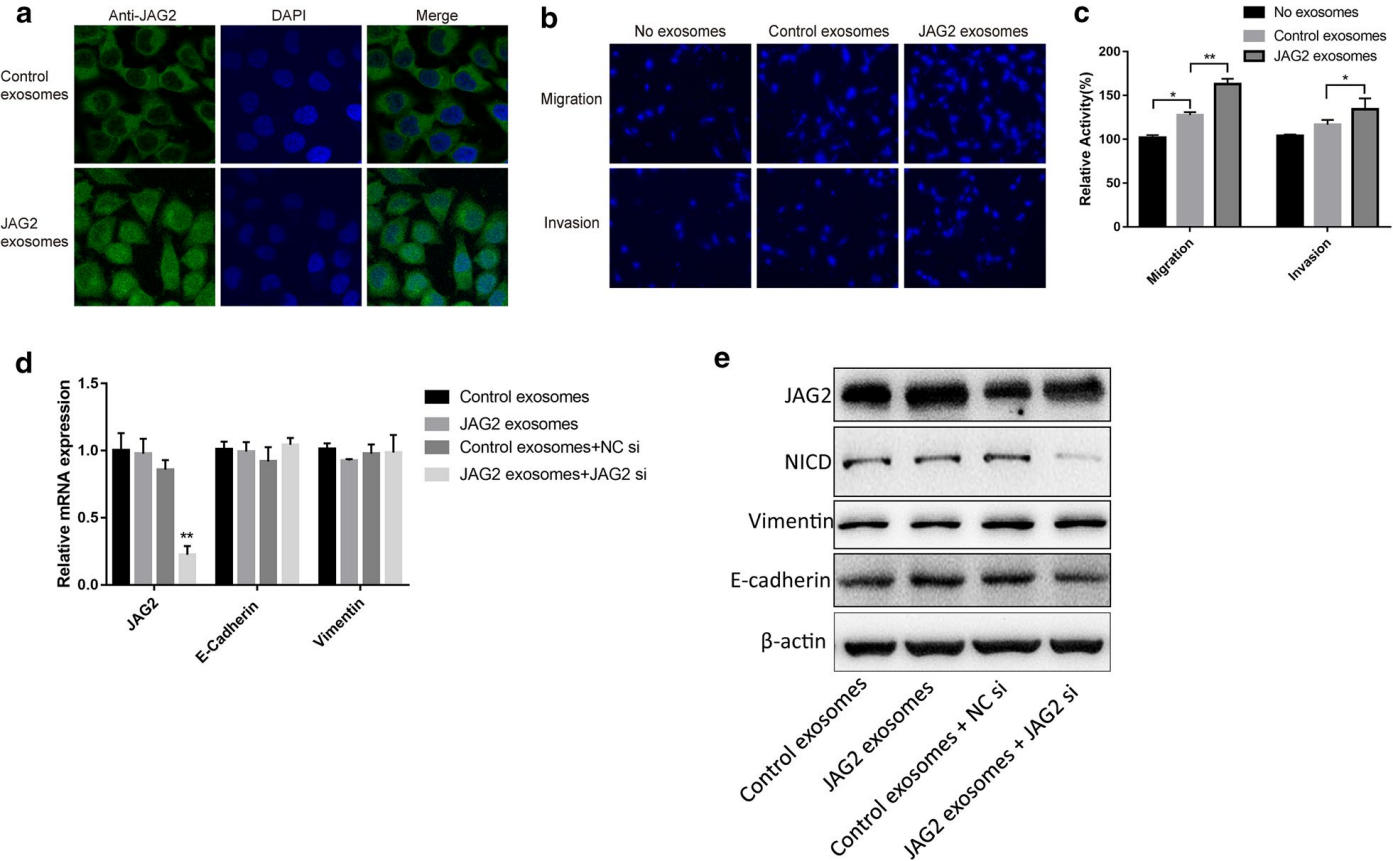
Migration and invasion of HT29 cells were promoted by JAG2-rich exosomes. **a** HT29 cells were incubated with control exosomes or JAG2-rich exosomes (40 μg/mL) for 24 h. Cells were then fixed and stained. Subcellular localization of JAG2 in HT29 cells was changed when treated with JAG2-rich exosomes; **b**, **c** migration and invasion of the HT29 cells were promoted when treated with JAG2-rich exosomes; **d**, **e** relative expression levels of related mRNAs and proteins were determined in JAG2-rich exosome-treated HT29 cells by real-time quantitative PCR and Western blot, respectively. Data are the mean ± SD. of three independent experiments. *P < 0.05 and **P < 0.01 versus control

**Fig. 9:**
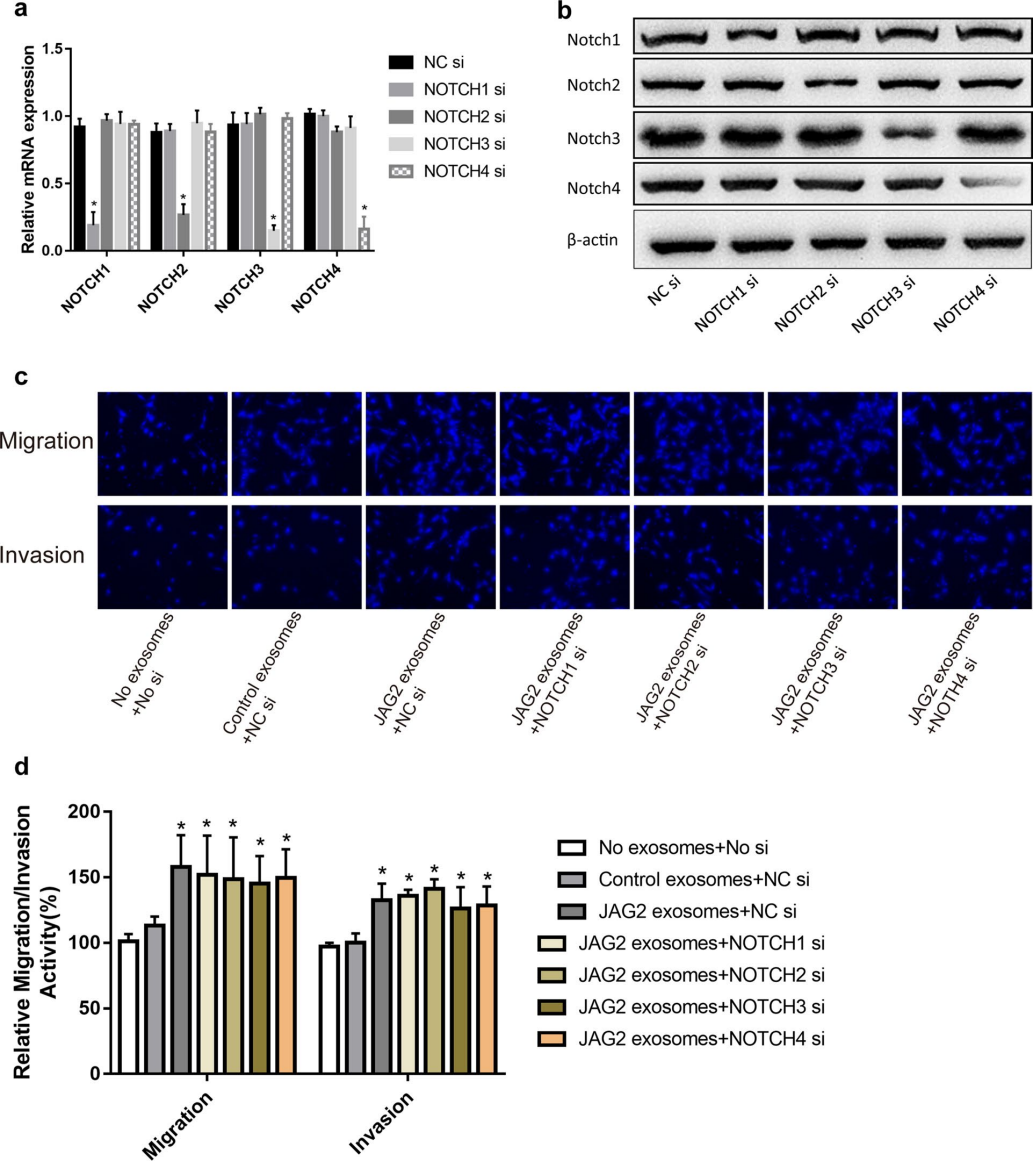
Correlation analysis between the migration/invasion-promoting effect of JAG2-rich exosomes and the Notch receptors. **a**, **b** Silencing of NOTCH1–4 by siRNA and the efficiency of siRNA silencing by real-time quantitative PCR and Western blot; **c**, **d** silencing NOTCH1–4 by siRNA could not effectively inhibit cell migration and invasion promoted by JAG2 exosomes. Data are the mean ± SD. of three independent experiments. *P < 0.05 versus control

**Fig. 10:**
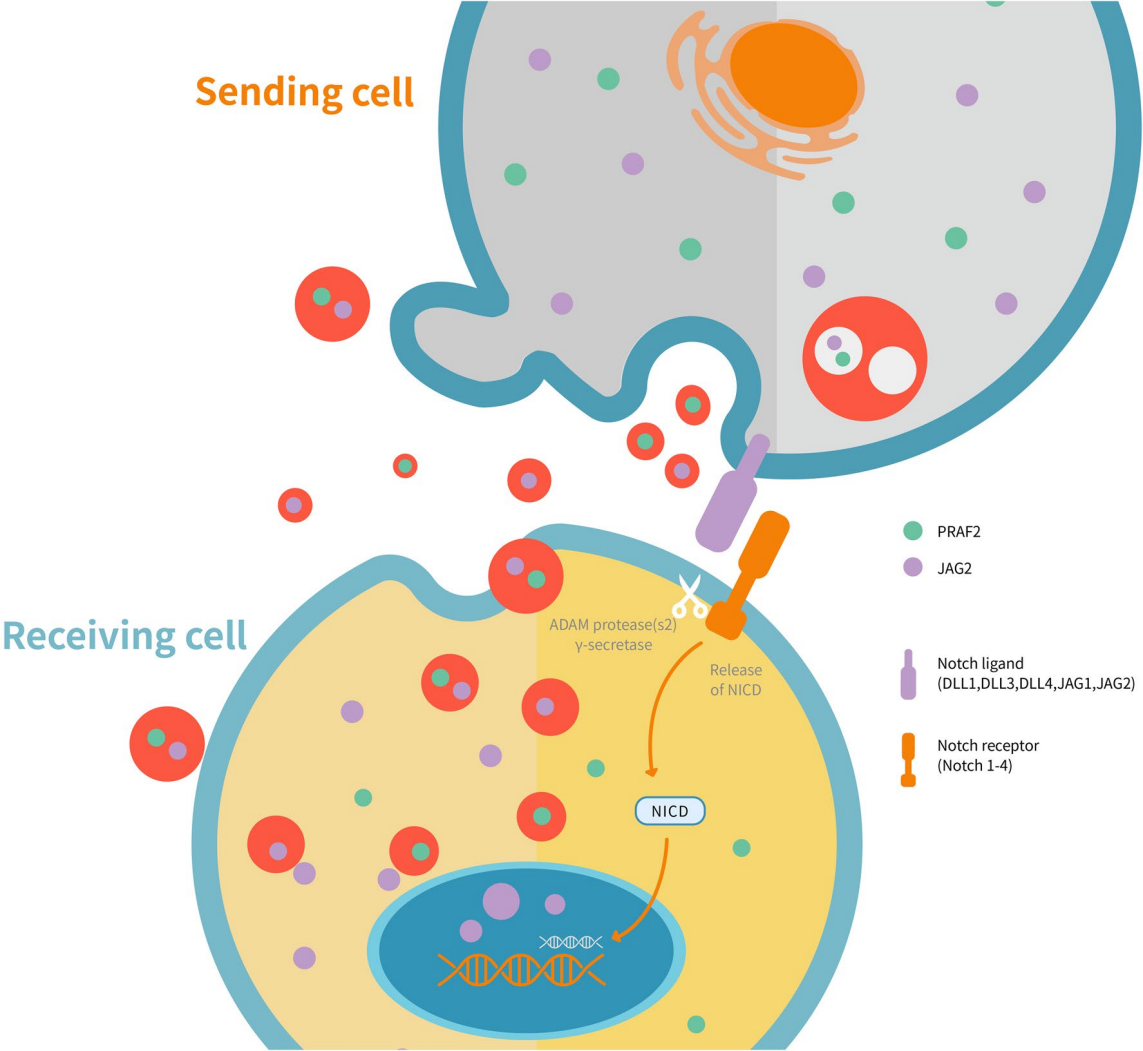
A hypothetical schematic model of the JAG2/PRAF2/Exosomes paracrine signaling promotes migration and invasion of colorectal cancer cells uncoupled from canonical Notch and EMT-dependent pathways

Exosomes from JAG2-overexpression HT29 cells were defined as JAG2-rich exosomes due to up-regulation of JAG2 protein content in exosomes. When normal HT29 cells were treated with these JAG2-rich exosomes, the subcellular localization of JAG2 varied. Most JAG2 proteins was distributed in the plasma membrane in the control group. However, JAG2 in the cells treated with JAG2-rich exosomes was uniformly distributed throughout the cells (Fig. 8a) and migration and invasion ability of these cells were significantly improved (Fig. 8b). Further examination revealed that intracellular JAG2 protein level was significantly upregulated in JAG2-rich exosome-treated HT29 cells, and no significant change in the level of JAG2 mRNA, however, was observed. The increase in JAG2 protein induced by exosomes could not be stopped even the endogenous JAG2 was reduced by siRNA (Fig. 8c, d). These results demonstrated that the increase in JAG2 protein level was contributed by the input of exogenous exosome. At the same time, no significant changes in the level of EMT markers (E-cadherin and vimentin) (Fig. 8d) and the JAG2-upregulated relative level of NICD were found, although cell migration ability was enhanced after JAG2 protein level was increased in cells when treated with exosomes. Next, Notch1–4 was pre-silenced by specific siRNAs, respectively, in HT29 cells (Fig. 9a, b) and then these cells were treated with JAG2-rich exosomes. The results showed that silencing of Notch1–4 did not prevent the enhancement of cell migration and invasion (Fig. 9c, d). Overall, the above results suggested that the paracrine effect of JAG2 in the form of exosomes regulated the metastatic ability of colorectal cancer cells, making it different from the canonical Notch and EMT signaling pathways (Fig. 10).

## Discussion

Current studies have shown that the Notch signaling pathway is closely related to tumor biological characteristics and is abnormally expressed in various tumors including colorectal cancer. High expression of the ligand JAG1, which is regulated by β-catenin, may upregulate the expression of Notch-1 in colorectal cancer, which persists throughout the process of tumor growth [20]. Abnormally increased Notch-1 inhibits tumor cell apoptosis and promotes the development and metastasis of tumors. Transcriptional repressor Kruppel-like factor (KLF4) inhibits the abnormally differentiated cells in colorectal cancer and HES-1, a downstream molecule of Notch-1, can inhibit the expression of KLF4 [21]. Notch-1 and Notch-2 function in opposite ways in colorectal cancer [22]. Notch-1 was negatively while Notch-2 was positively correlated with prognosis and therefore they play a completely different role in tumor formation and development. Notch-3 is highly expressed in metastatic carcinoma of colorectal cancer, suggesting its potential involvement in tumor metastasis [23]. In conclusion, both Notch receptors and ligands are abnormally expressed in colorectal cancer and Notch-3, Jagged-1 and Dll-4 are associated with tumor metastasis [24]. Dll-1 promotes the Wnt and transforming growth factor β (TGF-β) pathways and CTFG gene expression via binding to Smad2/3 and Tof-4, thereby promoting the development of colorectal cancer independent of Notch signaling pathways [25]. Notch also plays an active role in epithelial to mesenchymal transition (EMT) through synergistic effects with Slug, Snail, and TGF-β, enabling the mesenchymal phenotype for epithelial cells. Meanwhile, the expression of markers of interstitial cells is up-regulated and that of epithelial cells is down-regulated [26]. In endothelial cells, Notch-1 stimulates the expression of the Slug gene by inhibiting the expression of the transmembrane glycoprotein gene E-cadherin, which is responsible for the tight adhesion to adjacent cells, resulting in an EMT phenotype [27]. Fender et al. found that continuous stimulation of Notch1 may promote the expression of JAG1, Smad-3, Slug, and CD44 in colorectal cancer cell line HCT-116 [28]. It is speculated that the activated Notch-1 may first stimulate the expression of JAG1, which in turn activates Notch receptors such as Notch-3, to synergistically promote the expression of Slug and CD44 and ensure the EMT and stem cell phenotype. The important role of Notch signaling pathways in colorectal cancer stem cells has been widely confirmed now and the expression intensity of Notch in colorectal cancer stem cells is 10–30 times higher than that in common colorectal cancer cells. Notch plays an important role in the self-renewal of colorectal cancer stem cells and it prevents apoptosis by inhibiting transcription factors such as cell cycle regulatory proteins P27 and ATOH1 [29]. HES1 expression and consequently the number of stem cells and tumor progression can be reduced by inhibiting the expression of ligand DI1-4 in metastatic tumors. Molecules in the Notch signaling pathways, including HES1, Notch-1, and Jagged-1, are highly expressed in colorectal cancer stem cells [30]. Cell differentiation is induced by blocking the expression of HES1 with shRNAs interference and HES1 can inhibit cell differentiation in animal models. Therefore, Notch pathway plays an important role in regulating and maintaining the formation of cancer stem cells and the development of intestinal cancer. Moreover, the most fundamental and critical regulation of the Notch signaling pathway is to control the concentration of NICD in the nucleus.

Few studies on the function and mechanism of JAG2 in colorectal cancer have been reported to date. It was found in our previous studies that JAG2 was abnormally and highly expressed in colorectal cancer cells and that the expression level of JAG2 was positively correlated with the migration and invasion ability of colorectal cancer cells. The relative level of JAG2 in clinical specimens is also directly related to the clinical stages of disease. Traditionally, the most fundamental and critical regulation of the Notch signaling pathway is to control the concentration of NICD in the nucleus. However, it was shown in our cell model that JAG2 was involved in the regulation of migration and invasion rather than cell proliferation [16], and this effect was independent of NICD concentration and the reported Notch signaling pathway. More interestingly, JAG2 also promoted the migration and invasion of colon cancer cells in a non-EMT pathway. Further analysis revealed the co-expression of JAG2 with PRAF2 in colorectal cancer cells. JAG2-rich exosomes were released from colorectal cancer cells in a PRAF2-dependent way, while these exosomes regulated the metastasis of colorectal cancer cells in a paracrine manner. This is the evidence supporting the biological function of JAG2 through non-canonical Notch and non-EMT-dependent pathways and also the first demonstration of the functions of PRAF2 in colorectal cancer cells. In fact, Hu et al. [12] also reported that JAG2 promotes the migration and invasion of pancreatic ductal adenocarcinoma cells independent of activation of Notch downstream signal transduction. These results undoubtedly reveal that JAG2’s function in tumors has exceeded traditional cognition.

PRAF2 is a 19 kDa protein with four transmembrane domains and belongs to the Prenylated Rab acceptor 1 domain family (PRAF). Members of the PRAF family are structurally related and all contain a large PRA1 domain. PRAF protein is usually located in ER, Golgi and vesicle structure of cells [31]. Two other members of the PRAF protein family (PRAF1 and PRAF3) have been studied to some extent. Many of these studies have associated PRAF1 and PRAF3 with several well-known cancer signaling pathways. For example, PRAF1 (also known as RABAC1, PRA1, and YIP3) was initially found to interact with small GTPase proteins that regulate intracellular vesicular transport [32]. In addition, PRAF1 is involved in cell proliferation and tumorigenesis by binding to β-catenin to inhibit TCF/β-catenin signaling [33]. PRAF3 (also known as JWA, GTRAP3-18, and Aip-5) is a complete ER membrane protein upregulated by retinoic acid (RA) and then activated by excitatory amino acid vector 1 (EAAC1), a primary neuronal glutamate transporter [34]. PRAF3 is also defined as a potential inhibitor to inhibit the migration of cancer cells by regulating the MAPK signaling pathway and F-actin cytoskeleton [35].

However, research on PRAF2 is currently rare. PRAF2 was first discovered to be a protein that interacts with human chemokine receptor 5 (CCR5) and human phosphoinositide phosphodiesterase (GDE1/MIR16) [36]. Human PRAF2 is highly expressed in various tissues such as small intestine, lung, spleen and pancreas, and is most prominent in the brain [37]. Also, PRAF2 is overexpressed in breast, colon, lung, and ovarian tumor tissue specimens relative to normal tissue specimens [31, 38]. Yco et al. [39] found that PRAF2 could stimulate the proliferation and migration of neuroblastoma cells and predict poor prognosis. Wang et al. [40] revealed abnormal expression of PRAF2 in hepatocellular carcinoma tissues and demonstrated that PRAF2 could promote cell proliferation, migration and tumor metastasis by in vitro and in vivo experiments. Using UALCAN online tool (http://ualcan.path.uab.edu) to invoke relevant data in TCGA database, the analysis results showed that PRAF2 presented significantly high expression in colorectal cancer tissues (Additional file 1: Figure S1A). The abnormal expression of PRAF2 was revealed to be significantly correlated with the shortened overall survival of patients by using the Human Protein Atlas online tool analysis (https://www.proteinatlas.org) (Additional file 1: Figure S1B). Our experimental results reveal for the first time that PRAF2 overexpression could increase the secretion of colon cancer cell exosomes, and promote the migration and invasion of tumor cells in a JAG2-dependent manner through signal transduction in the microenvironment. On the other hand, knocking down PRAF2 significantly reduced the content of PRAF2 in exosomes, revealing that PRAF2 is involved as a component itself in the formation of exosomes.

However, as far as the preliminary results are concerned, there are still many mechanisms to be clarified. For example, why JAG2 is co-expressed with PRAF2, is JAG2’s positive regulation of PRAF2 direct or indirect, and does the regulation occur at the transcriptional level or the post-transcriptional level, which need to be explored further. At least we need to supplement the RNA sequencing analysis in the next experiment to explore the specific mechanism of co-expression of JAG2 and PRAF2. Additional information on questions such as does PRAF2 function in exosome secretion depend on Rab receptors, why JAG2 is co-expressed with PRAF2, how JAG2 is released into exosomes, and details of the exocrine effects of exosomes are not available now, which need to be addressed in further studies. Also, whether the biological function of JAG2 exhibited in the form of exosomes can be extrapolated to other tumor cells remains unknown.

Another question worth pondering is whether the inactivation of the EMT key protein itself is responsible for the independence of the JAG2/PRAF2 pathway from EMT. For example, E-cadherin is inactivated in tumor cells due to gene mutation, heterozygosity loss and DNA methylation. Fortunately, the results of Figs. 2c, 3a and 8e showed that CDH1 was not lost in HT29 cells. Subsequent sequencing analysis found only one synonymous mutation at the 2076 nucleotide of CDH1 mRNA (T → C, NM_004360.5) (Additional file 1: Figure S2). However, whether the epigenetic regulation of E-cadherin and Vimentin would affect the regulatory pathway of JAG2 and tumor metastasis and the universality of JAG2 related pathways are still worthy of further exploration.

## Conclusion

In summary, we revealed the unusual and interesting biological functions of JAG2 in colorectal cancer cells. This is the evidence supporting the biological function of JAG2 through non-canonical Notch and non-EMT-dependent pathways and also the first demonstration of the functions of PRAF2 in colorectal cancer cells. These findings also provide theoretical basis for the development of small molecules or biological agents for therapeutic intervention targeting JAG2/PRAF2.

## Additional file

**Additional file 1: Table S1.** Patients characteristics. **Table S2.** Mass spectrometry analysis of proteins co-expressed with JAG2 in HCT116 cells. **Table S3.** Summary of cell characteristics. **Figure S1.** A The expression of PRAF2 in different stages of colorectal cancer was analyzed by UALCAN database; B Kaplan-Meier survival plots demonstrating that high PRAF2 expression levels correlated with worse overall survival (OS) in colorectal cancer patients (n = 597) from PROTEINATLAS database. **Figure S2.** The presence of E-cadherin mutations and polymorphisms in HT29 cells.

## Data Availability

All data provide in the main paper or Additional file.

## Abbreviations

MTT: epithelial–mesenchymal transition
NECD: extracellular domain of Notch
LNRs: Lin-12/Notch repeats
NICD: Notch intracellular domain
TACE: TNF-α converting enzyme
CoR: corepressor
CoA: coactivator
DMEM: Dulbecco’s modified eagle medium
MEM: modified Eagle’s medium
QRT-PCR: quantitative real-time polymerase chain reaction
Nano-LC–MS/MS: nanoscale liquid chromatography coupled to tandem mass spectrometry
