# Supplementary material for: Mutual regulation of JAG2 and PRAF2 promotes migration and invasion of colorectal cancer cells uncoupled from epithelial–mesenchymal transition
Source: Cancer Cell Int. 2019 Jun 11;19:160. doi: 10.1186/s12935-019-0871-5 (PMC6558914; doi:10.1186/s12935-019-0871-5)
Supplement: Supplementary file 1 — Additional file 1: Table S1. Patients characteristics. Table S2. Mass spectrometry analysis of proteins co-expressed with JAG2 in HCT116 cells. Table S3. Summary of cell characteristics. Figure S1. A The expression of PRAF2 in different stages of colorectal cancer was analyzed by UALCAN database; B Kaplan-Meier survival plots demonstrating that high PRAF2 expression levels correlated with worse overall survival (OS) in colorectal cancer patients (n = 597) from PROTEINATLAS database. Figure S2. The presence of E-cadherin mutations and polymorphisms in HT29 cells. [file 12935_2019_871_MOESM1_ESM.docx]

**Additional Tables**

**Table S1. Patients Characteristics**

| **Characteristics** | Colorectal cancer (n=52) |
| --- | --- |
| **Gender** |  |
| Male | 30 |
| Female | 22 |
| **Age at diagnosis** |  |
| Mean±SD | 60±12 |
| Median (range) | 56 (32-81) |
| **Clinical stage** |  |
| Ⅰ | 9 |
| Ⅱ | 11 |
| Ⅲ | 32 |
| **Nodal status** |  |
| Positive | 32 |
| Negative | 20 |
| **Tumor location** |  |
| Rectum | 21 |
| Distal colon | 14 |
| Proximal colon | 17 |
| **Histology** |  |
| Adenocarcinoma | 45 |
| Mucous adenocarcinoma | 7 |
| signet-ring cell carcinoma | 0 |

**Table S2. Mass spectrometry analysis of proteins co-expressed with JAG2 in HCT116 cells**

| Protein symbol | Biological Functions | Human physiological processes or disease states involved |
| --- | --- | --- |
| KIF1B | ATPase activity and microtubule motor activity | Charcot-Marie-Tooth Disease, Axonal, Type 2A1 and Neuroblastoma |
| DDX39A | A member of the DEAD box protein family，a putative RNA helicase | Gastrointestinal Stromal Tumor and Inflammatory Bowel Disease 6 |
| TMEM223 | Transmembrane protein | Increase cell survival, transferrin endocytosis |
| RGPD4 | Intracellular transport and sorting protein family members | Asperger Syndrome |
| DIAPH2 | Interacts with the actin cytoskeleton to regulate the migration of early endosomes. | Premature Ovarian Failure 2A and Premature Ovarian Failure 1 |
| PRAF2 | Vesicle transport | Neuroblastoma |
| ATG4B | Autophagy Pathway | Progression of hepatocellular carcinoma |
| RBM33 | Regulate RNA cleavage, translation and transport | Cerebellar lesion |
| PTPN12 | Protein tyrosine phosphatase | Participation in the activation of EphA2 and EGFR pathways in colorectal cancer |
| DPY19L1 | Transferase activity, transferring glycosyl groups and mannosyltransferase activity | Regulates the radial migration of glutamatergic neurons in the developing cerebral cortex |
| ZNF593 | Nucleic acid binding and transcription corepressor activity | Negatively modulates the DNA binding activity of Oct-2 and therefore its transcriptional regulatory activity |
| RPL37 | Ribosomal Protein L37 | Viral mRNA Translation and Influenza Viral RNA Transcription and Replication |
| POLR2G | the seventh largest subunit of RNA polymerase II, responsible for synthesizing messenger RNA in eukaryotes | Myasthenic Syndrome, Congenital, 5 and Congenital Myasthenic Syndrome. |
| COL4A3BP | a kinase that specifically phosphorylates the N-terminal region of the non-collagenous domain of the alpha 3 chain of type IV collagen | Mental Retardation, Autosomal Dominant 34 and Goodpasture Syndrome |
| VKORC1 | The catalytic subunit of the vitamin K epoxide reductase complex, which is responsible for the reduction of inactive vitamin K 2,3-epoxide to active vitamin K in the endoplasmic reticulum membrane | Coumarin Resistance and Vitamin K-Dependent Clotting Factors, Combined Deficiency Of, 2. |
| LNPK | Endoplasmic reticulum (ER)-shaping membrane protein that plays a role in determining ER morphology | Involved in the stabilization of nascent three-way ER tubular junctions within the ER network |

**Table S3. Summary of cell characteristics**

| Cell line | Patient | Organ | Disease | Stage | Derived from | Reference | MSI status | CIMP panel | CIN | KRAS | BRAF | PIK3CA | PTEN | TP53 |
| --- | --- | --- | --- | --- | --- | --- | --- | --- | --- | --- | --- | --- | --- | --- |
| RKO |  | Colon | Colonic carcinoma |  | Primary tumor | Brattain et al.[1, 2] | MSI | + | - | wt | V600E | H1047R | wt | wt |
| HT29 | 44-Year-old female | Colon | Colorectal adenocarcinoma | Dukes’ C | Primary tumor | Fogh.[3] | MSS | + | + | wt | V600E | P449T | wt | R273H |
| HCT116 | 48-Year-old male | Colon ascendens | Colorectal carcinoma | Dukes’ D | Primary tumor | Brattain et al.[1, 4] and  Eshleman et al.[5] | MSI | + | - | G13D | wt | H1047R | wt | wt |
| SW620 | 51-Year-old male | Colon | Colorectal adenocarcinoma | Dukes’ C | Lymph node  metastasis | Leibovitz et al.[6] | MSS | + | + | G12V | wt | wt | wt | R273H;P309S |
| SW480 | 50-Year-old male | Colon | Colorectal adenocarcinoma | Dukes’ B | Primary tumor | Leibovitz et al.[6] | MSS | - | + | G12V | wt | wt | wt | R273H;P309S |
| DLD-1 | Male | Colon | Colorectal adenocarcinoma | Dukes' C | HCT-15/DLD-1  misclassified | Chen et al.[7] and Dexter et al.[8] | MSI | + | - | wt | V600E | wt | E157fs;R233X | wt |
| CCD-18co | 2.5  Months  female | Colon | Normal |  |  | Sugarman et al. [9] |  |  |  | wt | wt | wt | wt | wt |

Abbreviations: CIN, chromosomal instability pathway; MSI, microsatellite instability; MSS, microsatellite stable; CIMP, CpG island methylator phenotype; X, stop

codon; fs, frame shift; wt, wild type.

**Additional Figures**


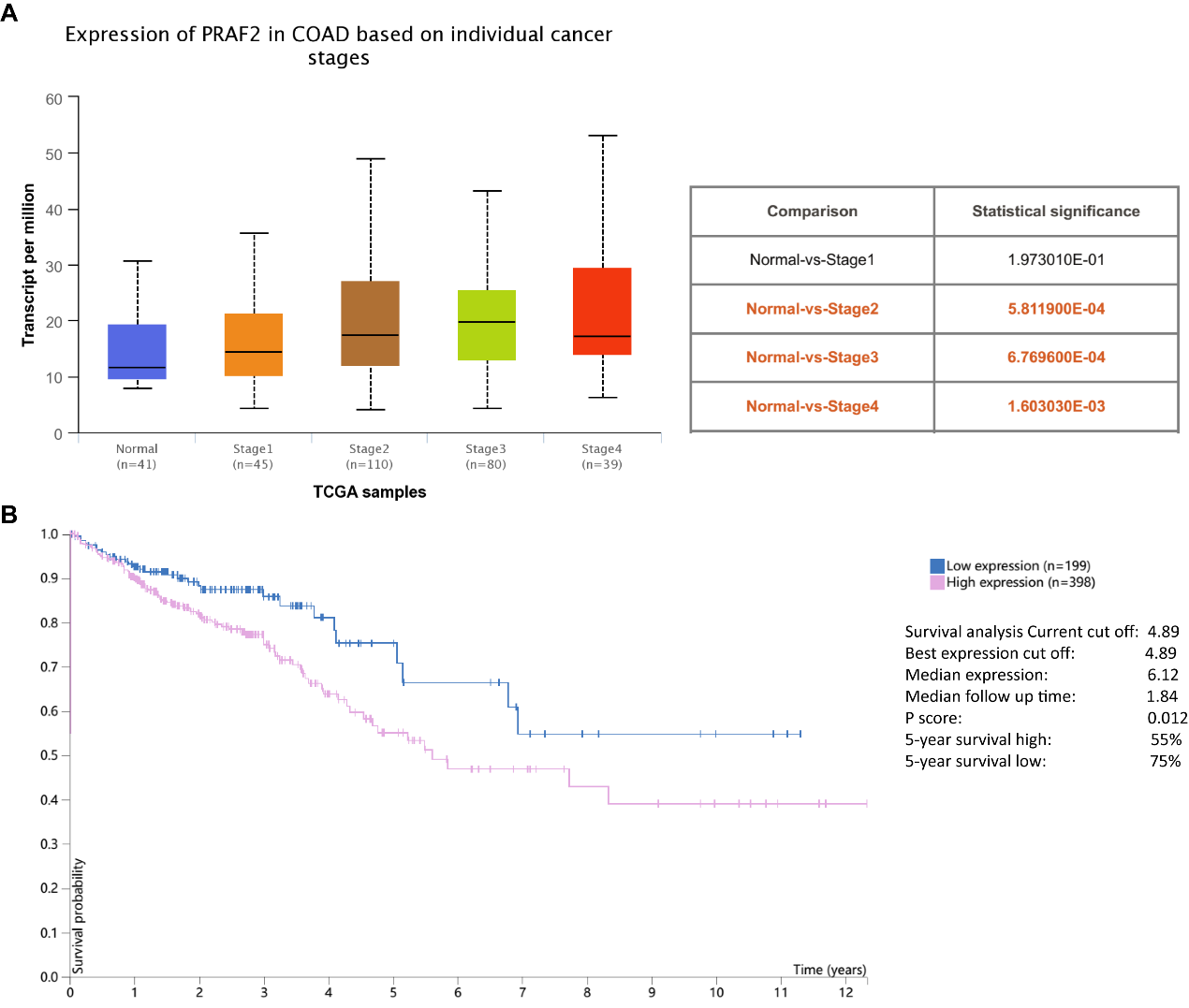


**Fig. S1** **A** The expression of PRAF2 in different stages of colorectal cancer was analyzed by UALCAN database; **B** Kaplan-Meier survival plots demonstrating that high PRAF2 expression levels correlated with worse overall survival (OS) in colorectal cancer patients (n=597) from PROTEINATLAS database.


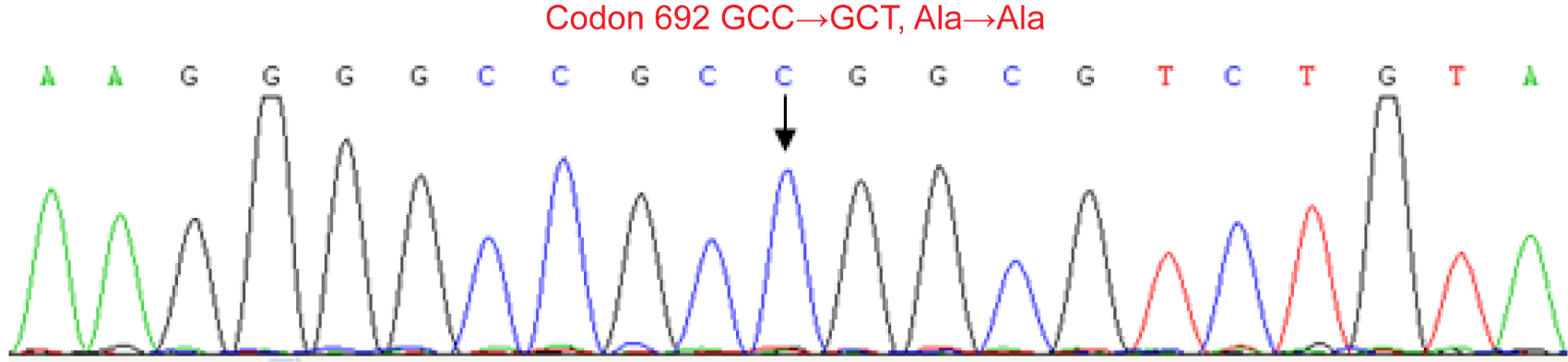


**Fig. S2** The presence of E-cadherin mutations and polymorphisms in HT29 cells.

**Additional** **References**

[1] Brattain MG, Brattain DE, Fine WD, Khaled FM, Marks ME, Kimball PM, Arcolano LA, Danbury BH. Initiation and characterization of cultures of human colonic carcinoma with different biological characteristics utilizing feeder layers of confluent fibroblasts. Oncodev Biol Med 1981; 2:355-366.

[2] Brattain MG, Levine AE, Chakrabarty S, Yeoman LC, Willson JK, Long B. Heterogeneity of human colon carcinoma. Cancer Metastasis Rev 1984; 3:177-191.

[3] Fogh J. Human tumor cells in vitro. 2013 .

[4] Brattain MG, Fine WD, Khaled FM, Thompson J, Brattain DE. Heterogeneity of malignant cells from a human colonic carcinoma. Cancer Res 1981; 41:1751-1756.

[5] Eshleman JR, Lang EZ, Bowerfind GK, Parsons R, Vogelstein B, Willson JK, Veigl ML, Sedwick WD, Markowitz SD. Increased mutation rate at the hprt locus accompanies microsatellite instability in colon cancer. Oncogene 1995; 10:33-37.

[6] Leibovitz A, Stinson JC, McCombs WB, McCoy CE, Mazur KC, Mabry ND. Classification of human colorectal adenocarcinoma cell lines. Cancer Res 1976; 36:4562-4569.

[7] Chen TR, Dorotinsky CS, McGuire LJ, Macy ML, Hay RJ. DLD-1 and HCT-15 cell lines derived separately from colorectal carcinomas have totally different chromosome changes but the same genetic origin. Cancer Genet Cytogenet 1995; 81:103-108.

[8] Dexter DL, Spremulli EN, Fligiel Z, Barbosa JA, Vogel R, VanVoorhees A, Calabresi P. Heterogeneity of cancer cells from a single human colon carcinoma. Am J Med 1981; 71:949-956.

[9] Sugarman BJ, Aggarwal BB, Hass PE, Figari IS, Palladino MA, Shepard HM. Recombinant human tumor necrosis factor-alpha: effects on proliferation of normal and transformed cells in vitro. Science 1985; 230:943-945.
